# How to address the body after breast cancer? A proposal for a psychological intervention focused on body compassion

**DOI:** 10.3389/fpsyg.2022.1085837

**Published:** 2023-01-09

**Authors:** Valeria Sebri, Ilaria Durosini, Gabriella Pravettoni

**Affiliations:** ^1^Applied Research Division for Cognitive and Psychological Science, IEO European Institute of Oncology IRCCS, Milan, Italy; ^2^Department of Oncology and Hemato-Oncology, University of Milan, Milan, Italy

**Keywords:** breast cancer, self, body image, injured self, body compassion

## 1. Introduction

Breast cancer is one of the most frequent cancers among women worldwide and strongly affects Quality of Life (Ferlay et al., 2015; Andreis et al., 2018). On a physical level, oncological treatments and interventions (e.g., surgery, chemotherapy, radiotherapy, and hormonal therapy) greatly impact the body (Serletti et al., 2011). Body image (BI) is conceptualized as an “internal representation of one's own outer appearance” (Thompson et al., 1999, p. 4). Specifically, BI does not refer only to the appearance on an aesthetical level but also to the mental representation of the body and its related emotions (Lewis-Smith et al., 2018; Sebri et al., 2020). Undesirable appearance-related side effects of breast cancer such as the loss of one or both breasts, hair loss, and scarring after surgical intervention have relevant psychological effects on BI and related wellbeing (Falbjork et al., 2013; Fioretti et al., 2017). Current research has highlighted the impact of surgical procedures (i.e., mastectomy with/without breast reconstruction and breast-conserving surgery) on BI (Collins et al., 2011; Chen et al., 2012). A systematic-review by Martins Faria et al. (2021) evidenced that mastectomy impairs BI strongly, in both the short and long-term. Particularly, radical breast removal decreases BI satisfaction in comparison to less radical procedures, such as breast- conserving surgery. In this regard, breast removal leaves a permanent and negative mark on the perception of the own body even after a cure. Similarly, Zhou et al. (2020) stated that many women experience BI disturbance after surgery due to the less body satisfaction. Accordingly, women with a history of breast cancer may perceive their body as a source of danger and fear. Interoceptive sensations, once ignored, suddenly become salient, with the risk of promoting the fear of cancer recurrence (Harris et al., 2017). This may be seen a behavioral level as women may perform “checking behaviors” such as excessive breast self-examinations daily (McGinty et al., 2016). To address these findings, the purpose of this contribution is to describe a proposal for a new psychological intervention focused on body compassion, which would promote compassion and BI to improve quality of life in women with a history of breast cancer.

## 2. Body, sexuality, and intimate relationships after cancer

Because of breast cancer treatment sexuality and intimate relationships can become difficult (Bishop, 2015), impacting the couple's life. Changes in the body and in the evaluation of women femininity allows for a perception of sexual unattractiveness (Sebri et al., 2021b). Moreover, oncological treatments can lead to the fear of losing fertility, which may impact monogamous relationships. Women rely on their partner to perform crucial roles of providing emotional support, managing finance, and making decisions, which can lead to high levels of distress (Akpor et al., 2022). Despite this, the partner is essential in the promotion the survivor's wellbeing, which serves to support the couple throughout the cancer journey. Literature shows the relevance of supporting marital adjustment to avoid excessive distress and anxiety (Suo et al., 2022) and strongly reduce negative BI (Akpor et al., 2022). BI impairments can also affect social relationships. Specifically, a negative BI may lead women to constantly scrutinize their body with the fear of being different from cultural stereotypes through the development of negative emotions (Sherman et al., 2018; Triberti et al., 2019). In line with the Self Discrepancy Theory by Higgins (1987), the higher the discrepancy between “whom they perceive to be” and “whom they would like to be,” the higher psychopathological outcomes and emotional issues could be observed. In this regard, impairments in emotional regulation can be observed in young women with a history of breast cancer especially (Miyashita et al., 2015). After oncological treatments, the risk of incurring premature menopause and consequently to the risk of infertility can change the perception of body and femininity, in particular for young women (Camp-Sorrell, 2009). Moreover, young women could experience the difficulty in managing their fear of not seeing their child/children grow up (Miyashita et al., 2015).

Starting from the background, women with a history of breast cancer tend to reframe their identity as that of a or “woman at risk,” leading to rethinking future expectations and taking health management into account (Gibson et al., 2015; McGannon et al., 2016; Moskalewicz et al., 2022). In this way, women need to deal with a renovated overall self, which is described as a system of cognitive and affective schemas that affects life-meanings and decisions (Christoff et al., 2011; Sui and Humphreys, 2015; Sebri et al., 2021c). Women tend to show a new self-representation, called *Injured Self*, an illness-schema rich with emotions and autobiographical memories related to the oncological journey, which is particularly relevant to create images of the self (Sebri et al., 2020). Thus, it is essential to integrate Injured Self and its related illness-memories into the overall self, by linking current self-representations, beliefs, and aims consistently to avoid self-fragmentation and promote wellbeing (Conway, 2005; Sebri et al., 2020).

Breast cancer patients and survivors need to integrate various self-schemas into a coherent one. For this aim, the episodic memory system provides input to the working self and includes some knowledge in autobiographical memory influenced by goal-relevance (Conway, 2005). This way, the working self is particularly important to create appropriate images of the Self following the self-coherence request (Markus and Nurius, 1986). During memory construction, the working self is indeed the moderator between the demands of memory (that corresponds to reality and actual experiences) and coherence (memory that should be consistent with one's current self-images, beliefs, and aims) (Conway et al., 2004).

The construction of a positive BI depends on the individual experience of the illness and on the possibility of addressing bodily issues and their related emotion to promote a new positive perception of the body after cancer. A recent meta-analysis showed the efficacy of novel and mixed-method psychological interventions (e.g., physical exercises, art therapy, and web-based interventions) to avoid self-fragmentation and promoting a positive BI, personal strength, and cognitive abilities (e.g., decision making and attention; Sebri et al., 2021a). The majority of the psychological interventions were focused on cognitive-behavioral/existential, interpersonal, psychosocial, supportive, emotionally expressive, and educational approaches (Blanco et al., 2014; Savioni et al., 2022). Additionally, recent studies highlighted the relevance of psychological interventions based on mindfulness and self-compassion intervention thanks to the promotion of kindness and lack of self-judgment, which are relevant to promote wellbeing in women with a history of breast cancer (Neff, 2003; Chang et al., 2021; Mifsud et al., 2021). Specifically, mindfulness-based stress reduction (MBSR) intervention allows women to have kindness and care toward the body specifically (Matchim et al., 2011).

## 3. The role of body compassion on injured self

Self-compassion is defined as the kindness toward the self and it is characterized by lack of self-judgment, acknowledging past trauma, and consideration of suffering as part of the human condition (Lazarus, 1991; Neff, 2003; Jazaieri et al., 2014). Kirby (2017) embodied this definition through their Compassion Cultivation Training. This program focused on promoting compassion toward others, in order to highlight positive outcomes, decrease suffering, and improve life satisfaction. When self-compassion is referred to the physical self on a cognitive, emotional, and behavioral level, it can be defined as body compassion (Altman et al., 2020). In other words, psychological interventions focused on body compassion have the goal of promoting attitudes of kindness and care with a specific focus on the body (Strauss et al., 2016; Van Niekerk et al., 2021). In accordance with it, a study by Matos et al. (2022) evidenced that the negative correlation between body compassion and emotional issues allows for the promotion of a positive BI.

In the breast cancer field, body compassion interventions can be relevant to promote positive emotions and attitudes toward the body after cancer (Sebri et al., 2022). Despite the efficacy of self-compassion interventions to promote BI (González-Hernández et al., 2021), there is a lack of understanding surrounding the efficacy of specific programs related to body compassion in women with a history of breast cancer. The purpose of this new psychological intervention based on body compassion and BI could be relevant to have a renovated perception of the body after cancer, focusing on attitudes of compassion toward the body and promoting wellbeing.

## 4. A body compassion intervention on BI: Contents and therapeutic aims

Based on the existing literature (e.g., Strauss et al., 2016; Kirby, 2017), we structured a new Body Compassion Intervention on Body Image to promote a positive BI in women with a history of breast cancer. According to Table 1, this psychological intervention will consist of four group sessions focused on recognizing negative emotions, accepting uncomfortable feelings, and promoting BI in women after cancer treatment. Specifically:

- **Session 1:** In the first session, the psycho-oncologist will introduce participants in taking care of the body after cancer with attitudes based on kindness and acceptance of their inner feelings. This aspect will help women to perceive their body not only as a source of danger and fear, but also as an ally. In fact, the intervention will introduce the possibility of a new and renovated body after cancer, with an integration of a new cancer-related self-representation, the **Injured Self**, in an overall and coherent self (Sebri et al., 2020). Furthermore, participants will be asked to share their motivations for participating in the group and their personal goals in a collaborative way (Durosini and Aschieri, 2021). Expected therapeutic aims will be a greater oncological adherence to treatments. Literature shows the relevance of patients' active role in their treatment and care (Castellano-Tejedor et al., 2015; Kondylakis et al., 2017).
- **Session 2:** In the second session, the psycho-oncologist will explain and introduce the mindfulness technique as an available tool in the psychological program to promote body awareness and general wellbeing. In particular, mindfulness-based stress reduction will improve participants' skills to recognize individuals' suffering and uncomfortable feelings related to the body, which generally increases the fear of cancer recurrence and emotional issues (AhmadiQaragezlou et al., 2020; Park et al., 2020). Specifically, women will be invited to be careful about their stressful sensation. Then, the psycho-oncologist will propose some strategies to manage distress and negative emotions, as suggested by the mindfulness-stress based reduction technique. Women will be invited to repeat the strategies indicated daily, especially when experiencing distress. This session will aim to associate BI and, in particular, each part of the body affected by cancer with positive feelings and emotions, decreasing negative sensations and feelings. Expected results will be the new possibility of thinking of the future and having positive expectations, adopting adaptive coping styles useful to face the Aisease (Zhang et al., 2010; Koch-Gallenkamp et al., 2016). This way, perceiving the body as a “helper” during everyday life challenges will help them to structure goal setting and future objectives (Sebri et al., 2022). After cancer, women may tend to perceive their body without physical energy and be unable to deal with daily challenges. This psychological intervention will aim to promote a positive perception of the body, which can sustain and manage everyday issues;
- **Session 3:** The third session will be focused on promoting bodily awareness by stressing the contact with the body and its sensations. In particular, women with a history of breast cancer will be more able to be aware of their inner sensations by reducing emotional arousal and promoting self-emotion regulation (Herwig et al., 2010). Moreover, the psycho-oncologist will introduce a discussion about common characteristics and shortcomings of humanity and recognize, elaborate, and accept the own body with its physical limits and difficulties. Breast cancer and its treatments change routines, relationships, and the lack of independence (Jacobs et al., 2018). In this session, the relevance of taking care will be highlighted. Therapeutic aims will focus on identifying and avoiding behaviors that cause stress. Participants will be accompanied in recognizing their needs and desire, without shame and fear of their inner sensations;
- **Session 4:** The last session will be focused on the practice of loving-kindness meditations conducted by the psycho-oncologist. The overall aim of this session will be focused on integrating the Injured Self into the overall Self by stressing bodily contact and its related emotions and, as a consequence, the acceptance of this new body after breast cancer (Sebri et al., 2020, 2022). Therapeutic aims will be based on reaching emotional regulation by promoting awareness of inner feelings and regaining control over them (Sebri et al., 2022).

**Table 1:** Sessions of the body compassion intervention on BI.

**The body compassion contents**	**Consequences on BI**	**Therapeutic aims in oncology**
SESSION 1 Set the mind to the possibility of loving the body after cancer. Explore the individual's motivation to act and alleviate suffering related to the own BI	Women are introduced to the idea of loving their body, shifting from the perception of an “ill body” to a new and renovated one after cancer	Introduce the possibility of a lovely body to increase behaviors of positive and active adherence to treatments as a personal choice
SESSION 2 Explain and develop mindfulness skills by recognizing suffering and uncomfortable feelings related to the body	BI can now be associated with the feeling of relaxation. Participants may think of their bodies by experiencing positive emotions, not only fear of cancer recurrence	Rethink to the future in terms of new possibilities and challenges sustained by a body that can be able to manage fatigue and daily distress
SESSION 3 Gain awareness and compassion toward the own body after cancer through embracing our shared common humanity	The body compassion program could sustain the idea of human frailty by promoting the acceptance of a body that needs care and treatments	- Gain awareness and acceptance of physical limits - Promote independence and acceptance of personal limitations, sustained by others' help
SESSION 4 Practice loving-kindness meditation sessions conducted by a psycho-oncologist with expertise in mindfulness-stress based reduction intervention	The overall change in BI can be now led to the mental representations of a new body, which can be not only a source of fear but cared	Promote introspection and awareness for inner feelings to increase emotional regulation

We recommended that this psychological program be conducted by a psycho-oncologist with an expertise in mindfulness-based stress reduction for oncological women (Kabat- Zinn, 1997; Segal et al., 2014).

## 5. Conclusion

Breast cancer may affect body perception deeply, with notable consequences on women with a history of breast cancer' quality of life (Lewis-Smith et al., 2018). The body after cancer is sometimes perceived as in constant need of support (Li et al., 2015; Rahmaningrum et al., 2020). Interoceptive and physical sensations lead to fear of cancer recurrence, negative emotions, and an illness-self schema (i.e., Injured Self; Sebri et al., 2020). All these aspects lead women to decrease their prospective thinking and goal setting, which are necessary for pursuing one's own personal future (Blanco et al., 2014). Promoting self-compassion in breast cancer patients is of paramount importance to promote kindness toward self and the body and to help the management of negative emotions. The present manuscript structured a new psychological intervention focused on body compassion that could help women to manage their emotional issues and promote a positive BI (Fafouti et al., 2010).

Limitations of the present contribution could be the generalization of this specific psychological program to other cancer populations due to the specific focus on breast cancer and its characteristics, which are strictly connected to femininity and body satisfaction. Future studies are required to better understand BI in people who received different oncological diagnoses and different interventions (e.g., mastectomy) to implement personalized interventions on women' needs. Future studies should still explore the other characteristics associated with chronic illness-related alterations to the overall self and the appropriate psychological interventions. It is also possible that body compassion has to be adapted to other chronic pathological conditions, focusing on the specificities of any disease. At the same time, future research should explore the efficacy of body compassion interventions with the integration of other activities, such as physical exercises, as sustained by literature (Beadle, 2020). For example, randomized control trials, longitudinal studies, and case studies are needed to support the present psychological program and assess its efficacy. It is needed to progress scientific investigation by capturing the causes, characteristics, and consequences of BI and Injured Self to tailor psychological interventions to women with a history of breast cancer' needs.

## Author contributions

VS conceived the ideas presented in the article and wrote the first draft. ID contributed with discussion on the ideas presented and edited the manuscript. GP contributed with important intellectual contents and supervised the whole process. All authors contributed to the article and approved the submitted version.
